# Kaposi sarcoma associated herpesvirus infection of primary human endothelial cells activates the proto-oncogene STAT3

**DOI:** 10.1186/1750-9378-7-S1-P38

**Published:** 2012-04-19

**Authors:** Christine A King, Craig McCormick

**Affiliations:** 1 Department of Microbiology and Immunology, SUNY Upstate Medical University, Syracuse, NY, USA; 2 Department of Microbiology and Immunology, Dalhousie University, Halifax, NS, Canada

## 

Kaposi’s sarcoma associated herpesvirus (KSHV) in the etiological agent for 3 AIDS-related cancers: Kaposi’s sarcoma (KS), primary effusion lymphoma, and multicentric Castleman’s disease. The molecular mechanisms used by KSHV to induce cancer are incompletely understood. KS lesions harbor proliferating latently-infected endothelial cells (ECs), large numbers of inflammatory cells, and marked neoangiogenesis. Considered the major driving force in the development of KS, these KSHV-infected ECs elaborate a variety of pro-inflammatory and angiogenic factors that contribute to tumourigenesis. Considerable evidence has accumulated suggesting a critical role for activated signal transducer and activator of transcription-3 (STAT3) in malignant transformation. STAT3 is a latent transcription factor that upon activation, drives the expression of a number of genes involved in cell proliferation, survival, and immune responses. Canonical STAT3 activation occurs via phosphorylation of Y705, dimerization, and nuclear translocation, followed by phosphorylation of S727 for maximal transcriptional activity. Activated STAT3 has been observed in a variety of malignancies and has been shown to induce fibroblast transformation *in vitro* suggesting that STAT3 is a proto-oncogene. Interestingly, evidence has accumulated suggesting a role for S727 mono-phosphorylated STAT3. Here we show that latent KSHV infection of primary human endothelial cells (ECs) *in vitro* activates STAT3, and identify a key latency protein, kaposin B, that contributes to this activation. Kaposin B expression in ECs causes STAT3 phosphorylation at S727, in the absence of significant Y705 phosphorylation, and enhanced expression of a subset of STAT3 target genes including CCL5. Recent work shows that the tripartite motif-containing protein 28 (TRIM28, a.k.a. TIF-1β, KAP-1) negatively regulates STAT3 by recruiting transcriptional silencing complexes. The repressive activity of TRIM28 is mediated by post-translational modifications and a key site in the regulation of repressor activity maps to S473. Phosphorylation of this residue disrupts the recruitment of transcriptional silencing complexes effectively deactivating the co-repressive function of TRIM28. Confocal microscopy and western blot analysis demonstrate phosphorylation of TRIM28 at S473 in KSHV latently infected and kaposin B expressing ECs. Taken together, our studies suggest kaposin B may contribute to tumourigenesis via constitutive activation of STAT3.

